# Blood Plasma TGF- β1 Concentration in Sporadic Dilatative Pathology of Ascending Aorta: More Questions than Answers

**DOI:** 10.1371/journal.pone.0129353

**Published:** 2015-06-23

**Authors:** Ramune Sepetiene, Vaiva Patamsyte, Giedrius Zukovas, Giedre Jariene, Zita Stanioniene, Rimantas Benetis, Vaiva Lesauskaite

**Affiliations:** 1 Institute of Cardiology of the Medical Academy, Lithuanian University of Health Sciences, Sukileliu 17, Kaunas, Lithuania; 2 Department of Cardiac, Thoracic and Vascular Surgery, Lithuanian University of Health Sciences, Eiveniu 2, Kaunas, Lithuania; UT-Southwestern Med Ctr, UNITED STATES

## Abstract

Transforming growth factor β1 (TGF- β1) is a cytokine that participates in a broad range of cellular regulatory processes and is associated with various diseases including aortic aneurysm. Increased TGF- β1 levels are associated with Marfan syndrome (MFS) caused by *FBN1* mutations and subsequent defects in signaling system. We studied TGF- β1 levels in 62 patients with sporadic, non syndromic, dilatative pathology of ascending aorta (DPAA) and in reference group subjects (n = 212). An initial screening of 212 reference individuals identified TGF- β1 gender discrepancies and age-dependent cytokine increase in women. Patients with DPAA had increased levels of TGF- β1 in comparison to reference group subjects (median 7.7 ng/ml, range 2.1–25.3, and median 6.2 ng/ml, range 1.0–33.1, respectively). There is a significant association between TGF-β1 concentration and DPAA (OR 1.084, CI 1.027–1.144, p = 0.004) but the mechanisms of cause and effect have not been established yet. Slightly increased TGF-β1 concentrations in patients with sporadic DPAA in comparison to the reference subjects show a potential use of TGF-β1 as a biomarker for the disease. However, cytokine dependence on age, gender, and other unknown factors among individuals with no cardiovascular complains reduces its specificity for DPAA. We would also like to raise awareness regarding the choice of methods when measuring TGF-β1 levels with an emphasis on preanalytical phase and the choice of sample.

## Introduction

Marfan syndrome (MFS) is caused by mutations in the fibrillin-1 encoding gene *FBN1*. It has been discovered that fibrillin-1 regulates the bioavailability of transforming growth factor –β1 (TGF-β1) [1]. The latter is a member of TGF- β pluripotent cytokine family involved in tissue morphogenesis and homeostasis [2]. Moreover, an altered TGF-β signaling contributes significantly to the pathology of MFS [1]. In humans, circulating total TGF- β1 concentrations are elevated in patients with MFS in comparison to control individuals; in addition, correlation between circulating TGF-β1 levels and aortic root diameters in MFS mutant *Fbn1*(C1039G/+) and wild-type mice is observed [3]. Recent studies demonstrate that Smad-independent TGF-β signaling has an essential function in aneurysm morphogenesis in MFS mice [4].

Hillebrand et al. (2014) demonstrated that TGF-β1 increase was observed not only in MFS patients, but also in other genetic aortic syndromes [5]. Considering the role of *FBN1* polymorphisms for sporadic thoracic aneurysm and dissection [6,7] we analysed TGF-β1 concentrations in blood plasma from patients with sporadic dilatative pathology of ascending aorta and reference group subjects.

## Methods

### Study subjects

The study included 62 patients (median age 59 years, range 27–79) operated at the Department of Cardiac, Thoracic and Vascular Surgery of the Lithuanian University of Health Science (LUHS) in 2013–2014 years due to (a) ascending aortic aneurysm (AAA) (n = 35), (b) post-stenotic dilatation (PSD) of the ascending aorta due to aortic valve stenosis (n = 21), and (c) Stanford A dissection (SAD), acute event (n = 6). Clinical characteristics of study subjects are presented in Table 1. PSD group featured 19 individuals (90%) with bicuspid aortic valve, one individual (5%) with a pseudobicuspid valve and the remaining one (5%) with a tricuspid aortic valve. The diagnosis was confirmed by transthoracic echocardiography before the surgery and/or by direct inspection during surgical repair. Clinical information on concomitant or intercurrent diseases was assessed by reviewing each patient’s medical history. Only 11% of the patients had no other apart from DPAA. Most commonly reported concomitant or intercurrent diseases are presented in Table 1. Histopathological investigation was carried out on all aortic specimens obtained during surgery. Exclusion criteria were phenotypic features of Marfan syndromes, aortitis and ulcerating atherosclerosis of the ascending aorta.

**Table 1:** Concomitant or intercurrent diseases in patients with dilatative pathology of ascending aorta (DPAA) and in Reference group subjects.

Concomitant or intercurrent diseases	DPAA (n = 62), n (%)			Reference group (n = 212), n (%)
	SAD n = 6	PSD n = 21	AAA n = 35	
Age (years)	54 (46–59)	60 (27–75)	59 (30–78)	62 (23–87)
Gender	M = 4 (66.7%)	M = 15 (71.4%)	M = 30 (85.7%)	M = 105 (49.5%)
	F = 2 (33.3%)	F = 6 (28.6%)	F = 5 (14.3%)	F = 107 (50.5%)
Coronary artery disease	2 (33.3%)*	11 (52.4%)*	14 (40.0%)*	1 (0.4%)
Hypertension	4 (66.7%)*	14 (66.7%)*	30 (85.7%)*	71 (31.3%)
Atrial fibrillation	2 (33.3%)*	2 (9.5%)*	6 (17.1%)*	-
Diabetes mellitus	-	1 (4.8%)*	-	1 (0.4%)
Urinary system pathology	-	-	4 (11.4%)*	2 (0.9%)
Gastrointestinal and biliary system pathology	1 (16.7%)*	3 (14.3%)*	9 (25.7%)*	7 (3.1%)
Locomotion system pathology	-	-	-	6 (2.6%)
Respiratory system pathology	1 (16.7%)*	-	1 (2.9%)*	2 (0.9%)

*percentage is calculated for each subgroup cases

SAD—Stanford A dissection; PSD—Post Stenotic Dilatation; AAA—Ascending Aortic Aneurysm

A reference group was recruited from a randomised cluster sampling of local population (n = 212). One cluster consisted of students and staff volunteers from LUHS (n = 72, median age 49 years, range 23–67) and the second cluster consisted of volunteers (n = 140, median age 65 years, range 41–87) appointed to their physician in Dainavos outpatient’s clinic (Kaunas). All volunteers completed questionnairs on the state of their health. Overall majority (63.9%) had no complains about their health state. Most commonly reported diseases are presented in Table 1. Neither of the included subjects reported having cancer or autoimmune diseases, associated with increased TGF-β1 level.[8]

Ethical approval was obtained from the Ethics Committee of the Lithuanian Health Science University (No: BE-2-12) and all subjects provided written informed consent for participation in the study.

### TGFß1 quantitative detection

Blood samples from DPAA patients were obtained before the surgery and our reference group gave blood at the time of arrival. Blood was collected using 3ml Venosafe blood collection tubes containing 5.9 mg K2EDTA (Terumo Europe, Belgium). Plasma was obtained within 2 hours by spinning for 15 min at 2500 rpm, aliquoted and stored frozen at -20°C. Plasma samples were tested in duplicate using eBioscience Platinum (Bender Med Systems GmbH, Austria) human TGF-β1 ELISA kit based on standard sandwich enzyme-linked immune-sorbent assay technology according to manufacturers’ instructions. Absorbance was measured using Stat-fax 4200 microplate reader using 450 nm as a primary wave length. Manufacturers indicated cross reactivity with TGF-β2 and TGF-β3 was less than 0.01%.

### Statistical analysis

Non-parametric Kruskal-Wallis test was used for statistical analysis. Spearman's rank order correlation was used to evaluate the effects of age and gender on TGF-β1 levels. The association of TGF- β1 concentration with DPAA was assessed using logistic regression model adjusted for gender and age. Statistical significance was set at p = 0.05.

## Results

**Fig 1:**
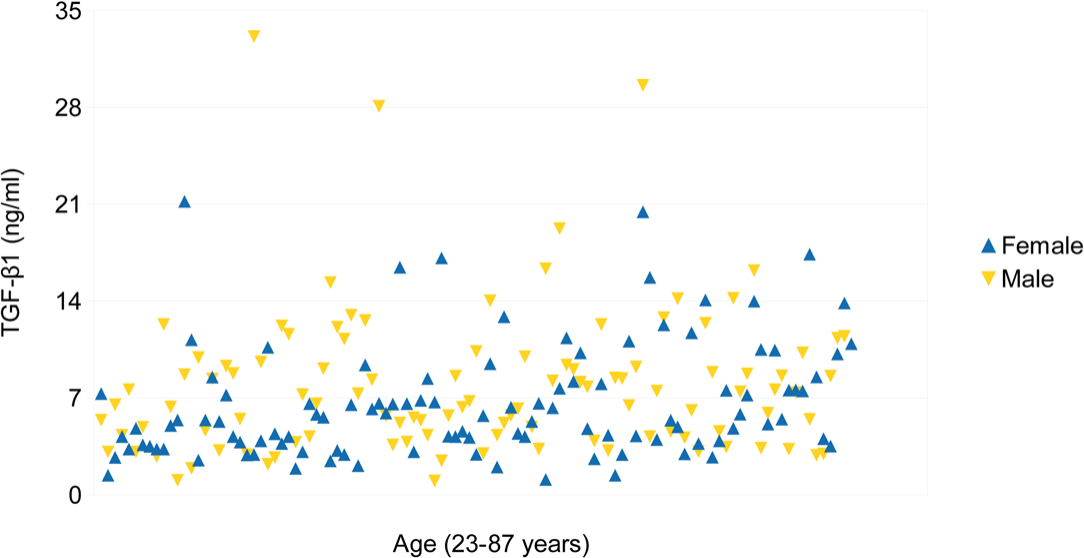
TGF-β1 concentration in the reference group according to the gender and age.

The reference group (n = 212) consisted of 105 males and 107 females (Table 1). Overall distribution of TGF-β1 values is shown in Fig 1. The lowest TGF-β1 concentration was 1.0 ng/ml and the highest was 33 ng/ml. Coefficient variation of results did not exceed the 4% detection limit of human TGF-β1 determined to be on average 8.6 ng/ml by six independent assays (Human TGF-β1 platinum ELISA product information and manual). Investigation of the reference group indicated higher TGF-β1 concentration (p = 0.017) in males (median 7.27 ng/ml, range 1.00–33.12) compared to females (median 5.38 ng/ml, range 1.10–21.20) (Table 2). TGF-β1 level showed a tendency to increase with age in women (R = 0.338, p<0.001) but was steady in men (R = 0.105, p = 0.288) throughout lifetime. These results indicate that age and gender can be confounding effects when comparing TGF-β1 levels between the study groups.

**Table 2:** Age and gender distribution among DPAA and Reference groups.

Group	Male	Female	Age (yrs) median (range)
DPAA (N = 62)	49	13	59 (27–79)
Reference (N = 212)	105	107	62 (23–87)

DPAA—Dilatative pathology of ascending aorta

Patients with Stanford A dissection had slightly higher TGF-β1 concentration, but there were no significant differences in TGF-β1 concentration between patients with various phenotypes of DPAA or between each phenotype and the reference group subjects. Overall, patients with DPAA had significantly higher TGF-β1 concentration than the reference group (Table 3). Differences in TGF-β1 values between DPAA males (median 7.60 ng/ml) and the reference male population (median 7.27 ng/ml) were not statistically significant (p = 0.135) whereas concentration between females in DPAA (median 8.80 ng/ml) and the reference group (median 5.38 ng/ml) were confidently higher (p = 0.004). Finally, we analyzed association between TGF-β1 concentration and DPAA which resulted in odds ratio (OR) 1.084 (95% confidence interval 1.027–1.144, p = 0.004).

**Table 3:** TGF-β1 concentration in patients with variuos phenotypes of dilatative pathology of ascending thoracic and reference group subjects.

TGF-β1	Stanford A dissection, n = 6	Post stenotic dilatation, n = 21	Aneurysm n = 35	Total n = 62	Reference group, n = 212
Median range) ng/ml	10.1 (6.58–17.6)	7.7 (2.1–22.0)	7.8 (2.3–25.3)	7.7 (2.1–25.3)*	6.2 (1.0–33.1)

*p = 0,010 versus Reference group

## Discussion and Conclusions

Recent studies draw attention to the role of TGF-β1 signaling system for aortic dilatation in MFS [3] or other genetic aortic syndromes [5]. We analyzed blood plasma TGF-β1 concentration in patients with sporadic dilatative pathology of ascending aorta and in the reference group subjects. For the first time we reported irregularity of TGF-β1 concentration in blood plasma caused by age and gender in subjects from the reference group. TGF-β1 concentration in males did not change with age and was significantly higher in comparison to females, while TGF-β1 concentration in females showed a significant increase due to older age. The latter phenomenon might be attributed to TGF-β1 signaling system activation by follicle-stimulating hormone (FSH) [9, 10]. It is well known that levels of FSH in females increase at perimenopausal age and decline only in the eighth/ninth decade [11]. FSH regulates post-menopausal endometrial atrophy by blocking TGF-β1 receptor II and blocking Smad2/3 activation pathway. FSH inhibitory action could compromise the ability of TGF-β1 to enter a cell and lead to an accumulation of TGF-β1 in blood plasma [9].

We gathered information on TGF-β1 concentrations in Marfan syndrome patients with DPAA and controls from previous studies and summarized it in Table 4. Higher TGF-β1 concentrations were reported in studies where blood serum was used. This phenomenon could be attributed to the presence of platelets which contain β-granules rich in TGF-β1 [12]. TGF-β1 concentration in serum accounts for both, the amount of cytokine in the systemic circulation and the amount released from platelet α-granules during clotting process [13]. TGF-β1 concentration detected in serum should be referred critically due to the strong positive correlation between platelet count and the serum concentration of TGF-β1 [14]. TGF-β1 variation among studies (Table 4) illustrates the importance of the blood sample used for testing and should always be taken into account when comparing results from different studies.

**Table 4:** Data on TGF-β1 concentration in patients with DPATA and controls.

Marfan Syndrome	Control group	Plasma/Serum	Assay/kit	Reference
43.78 ng/ml in dissection group	25.7 ng/ml	Serum	R&D Systems, Minneapolis, USA	Agg *et al*., 2014 [12]
31.64 ng/ml in annuloaortic ectasia group				
15 ng/ml in MFS without cardiovascular therapy	2.5 ng/ml	Plasma	R&D Systems, Minneapolis, USA	Matt *et al*., 2009 [3]
44.4 ng/ml	Without genetic aortic syndrome 32 ng/ml	Serum	R&D Systems, Minneapolis, USA	Hillerbrand *et al*., 2014 [5]
109 pg/ml	54 pg/ml	Plasma (?)	Bio-Rad, Richmond, Canada	Franken *et al*., 2013 [13]
1.31 ng/ml	1.17 ng/ml	Plasma	Mesco Scale Discovery, Gaithesburg, MD, USA	Ogawa *et al*., 2012 [14]

Our estimation of circulating TGF-β1 levels can be compared with the results reported by Matt and colleagues [3] since both studies used human blood plasma to obtain measurements. We obtained higher TGF-β1 values in the reference group compared to the latter study which could be attributed to the selection of the study subjects. Our reference group was three times larger and over a third of participants reported their health complains (Table 1). Some individuals with extremely high TGF-β1 values were not excluded from further analysis in order to evaluate TGF-β1 specificity in a real-life situation. This could partially explain why the cytokine values in our reference group were higher than the ones reported by Matt et al. [3].

Studies using both human blood plasma and serum indicate increased TGF-β1 values in Marfan patients with DPAA [3, 5, 14, 15]. We show that non-marfanoid patients with DPAA also have elevated plasma levels of TGF-β1 compared to the reference group. Patients with Stanford A dissection have particularly high TGF-β1 concentrations in comparison to patients with other phenotypes of DPATA. Such an increase could be caused by the liberation of TGF-β1 into the plasma from activated platelets during acute aortic rupture. Thus, we conclude that platelet activation might have an impact on the TGF-β1 concentration in blood plasma.

Standard methodical steps are used when measuring TGF-β1 concentration including acid activation and neutralisation with subsequent incubation and washing application to obtain active TGF-β1. Unfortunately, none of the commercially available tests have been evaluated by standardised method comparison methodology [16]. To avoid misevaluation of cytokine levels strict preanalytical procedures are recommended: (i) special venipuncture without a tourniquet [17] and (ii) detection of markers for platelet degranulation (e.g. platelet factor 4) simultaneously with TGF-β1 [12, 18] in order to reduce errors in preanalytical phase. The choice of experimental design could explain the discrepancies among the results reported in the Marfan studies (Table 4). Our study limitation is the fact that we did not carry out simultaneous measurements of markers for platelet degranulation so the effect of platelet activation on TGF-β1 concentration in blood plasma was not controlled analytically. Secondly, patients with DPAA had high prevalence (89%) of concomitant or intercurrent diseases which also might have an impact on higher levels of TGF-β1.

We reported a significant association between TGF-β1 concentration and DPAA but the mechanisms of cause and effect have not been established yet. Slightly increased TGF-β1 concentration in patients with sporadic DPAA in comparison to the reference subjects requires further investigation to establish its role in sporadic DPAA pathogenesis. We would also like to raise awareness regarding the choice of methods when measuring TGF-β1 levels with an emphasis on preanalytical phase and the choice of sample.
